# *Salvia miltiorrhiza* Bge. (Danshen) in the Treating Non-alcoholic Fatty Liver Disease Based on the Regulator of Metabolic Targets

**DOI:** 10.3389/fcvm.2022.842980

**Published:** 2022-04-22

**Authors:** Jie Liu, Yun Shi, Daiyin Peng, Lei Wang, Nianjun Yu, Guokai Wang, Weidong Chen

**Affiliations:** ^1^School of Pharmacy, Anhui University of Chinese Medicine, Hefei, China; ^2^Anhui Province Key Laboratory of Chinese Medicinal Formula, Hefei, China; ^3^Institute of Traditional Chinese Medicine Resources Protection and Development, Anhui Academy of Chinese Medicine, Hefei, China; ^4^Anhui Province Key Laboratory of Traditional Chinese Medicine Decoction Pieces of New Manufacturing Technology, Hefei, China

**Keywords:** Danshen, non-alcoholic fatty liver disease, metabolic targets, pharmacokinetic, molecular mechanism

## Abstract

Non-alcoholic fatty liver disease (NAFLD) is rapidly prevalent due to its strong association with increased metabolic syndrome such as cardio- and cerebrovascular disorders and diabetes. Few drugs can meet the growing disease burden of NAFLD. *Salvia miltiorrhiza* Bge. (Danshen) have been used for over 2,000 years in clinical trials to treat NAFLD and metabolic syndrome disease without clarified defined mechanisms. Metabolic targets restored metabolic homeostasis in patients with NAFLD and improved steatosis by reducing the delivery of metabolic substrates to liver as a promising way. Here we systematic review evidence showing that Danshen against NAFLD through diverse and crossing mechanisms based on metabolic targets. A synopsis of the phytochemistry and pharmacokinetic of Danshen and the mechanisms of metabolic targets regulating the progression of NAFLD is initially provided, followed by the pharmacological activity of Danshen in the management NAFLD. And then, the possible mechanisms of Danshen in the management of NAFLD based on metabolic targets are elucidated. Specifically, the metabolic targets c-Jun N-terminal kinases (JNK), sterol regulatory element-binding protein-1c (SREBP-1c), nuclear translocation carbohydrate response element–binding protein (ChREBP) related with lipid metabolism pathway, and peroxisome proliferator-activated receptors (PPARs), cytochrome P450 (CYP) and the others associated with pleiotropic metabolism will be discussed. Finally, providing a critical assessment of the preclinic and clinic model and the molecular mechanism in NAFLD.

## Introduction

NAFLD refers to a spectrum of liver diseases, which defined by steatosis in more than 5% of hepatocytes with little or no alcohol consumption (1). NAFLD is composed of non-alcoholic fatty liver (NAFL) and non-alcoholic steatohepatitis (NASH), which is characterized by steatosis, hepatocellular ballooning and lobular inflammation and accompanied by varying degrees of liver fibrosis (2). With the worsening of the fibrosis, some patients with NASH deteriorate to hepatic cirrhosis or even hepatic carcinoma. Currently, almost 25% of the general population worldwide suffer from NAFLD, and 20% of them will progress to hepatocirrhosis as the disease deteriorates (3). NAFLD is a metabolic stress liver injury, and its main pathogenesis is insulin resistance and lipid metabolism disorders. Metabolic syndromes such as obesity, insulin resistance, hyperglycemia, dyslipidemia, and hypertension are the main risk factors for NAFLD. However, drugs that interfere with NASH have side effects such as pioglitazone for insulin resistance, vitamin E for antioxidants, (4), and the only approved treatment is hygiene and dietary measures to date. At present, the prescription of traditional Chinese medical (TCM) contribute to the improvement of lipid infiltration of the liver and of the related anthropometric, and biochemical parameters (5). Besides, TCM may be promising treatment strategies for NAFLD due to their relatively cost-effective, multiple targets and few side effects.

Danshen has been used widely to treat metabolic syndromes such as hypertension, dyslipidemia, and hyperglycemia, which is consisted of active constituents such as hydrophilic phenols, lipophilic diterpenoids and polysaccharides (6). Accumulating evidence has suggested that the preventive and therapeutic potential of Danshen on NAFLD is related to reducing the risk of metabolic disorder in preclinic and clinic trials (7–10). What counts is, the frequency of clinical use of Danshen reached 7.28% in treatment with NAFLD, second only to the peach kernel (11). A case in point is that 8 randomized controlled trials with 800 patients of NAFLD showed that Danshen relieved the degree of hepatosteatosis by significantly reducing the plasma levels of transaminases (9).

The pathogenic driver of NAFLD is that the imbalance between glucose and lipid metabolism leads to excessively accumulated lipids within the liver (12, 13). Metabolic targets can rebalance the metabolic disorders and improve steatosis by reducing the delivery of metabolic substrates to the liver in patients with NAFLD (14–16). Additionally, a large number of pharmacological studies have shown that drugs targeting metabolic targets can effectively treat NASH, which proves that metabolic targets would be a promising prospect therapy for the treatment of NAFLD (4, 17, 18). Previous reviews discussed the prominent metabolic targets of NAFLD pharmacotherapy, including lipid metabolism pathway modulator acetyl-CoA carboxylase (acetyl-CoA), nuclear receptors, thyroid hormone receptors, glycemic modulator, sodium-glucose co-transporter 2 and fibroblast growth factors, etc. (12, 19). Herein we focus primarily on the significant metabolic targets associated with Danshen, including c-Jun N-terminal kinases (JNKs), SREBP-1c, ChREBP, PPARs, CYPs and the others, to provide theoretical basis for the development of a new way of Danshen treatment of NAFLD.

## Phytochemistry of Danshen

We reviewed phytochemistry of Danshen to fully understand its roles in NAFLD. Over 200 chemical components including phenolic acids, diterpenoids and polysaccharides have been identified from this plant (20).

The phenolic acids of Danshen contain a core skeleton of phenylpropanoid (C6-C3). It is generally believed that the phenolic acids are mostly conjugated with (R)-3- (3,4-Dihydroxyphenyl)-2-hydroxy- propanoic acid and derivatives or dimer of caffeic acid (21). For instance, Salvianolic acid A (SAA) is composed of tanshinol (TSL) and two molecules of caffeic acid. Previous studies have clarified the phenolic acids bioactivities such as anti-inflammatory, anti-oxidative, cardio-protection activity (22).

As a representative of diterpenoids, tanshinones are comprised of naphthalene or tetrahydronaphthalene rings A and B, ortho- or para-naphthoquinone or lactone ring C, and a furan or dihydrofuran ring D (21). Among these, tanshinone IIA (TSIIA) and cryptotanshinone (CT), which are characterized by an ortho-quinone C-ring. TSIIA, CT, tanshinone I (TSI), and dihydrotanshinone I; (TI) as primary and marker constituents in the official Chinese Pharmacopoeia, which exhibit the biological activity of anti-oxidative, anti-inflammatory, and anti-diabetic (23).

Besides, polysaccharides, flavonoids, steroids, and phenanthrenequinone have been identified in Danshen, which proved that a variety of pharmacological activity. For instance, the polysaccharides of Danshen exhibits immunomodulatory and antitumor effects (21).

**FIGURE 1:**
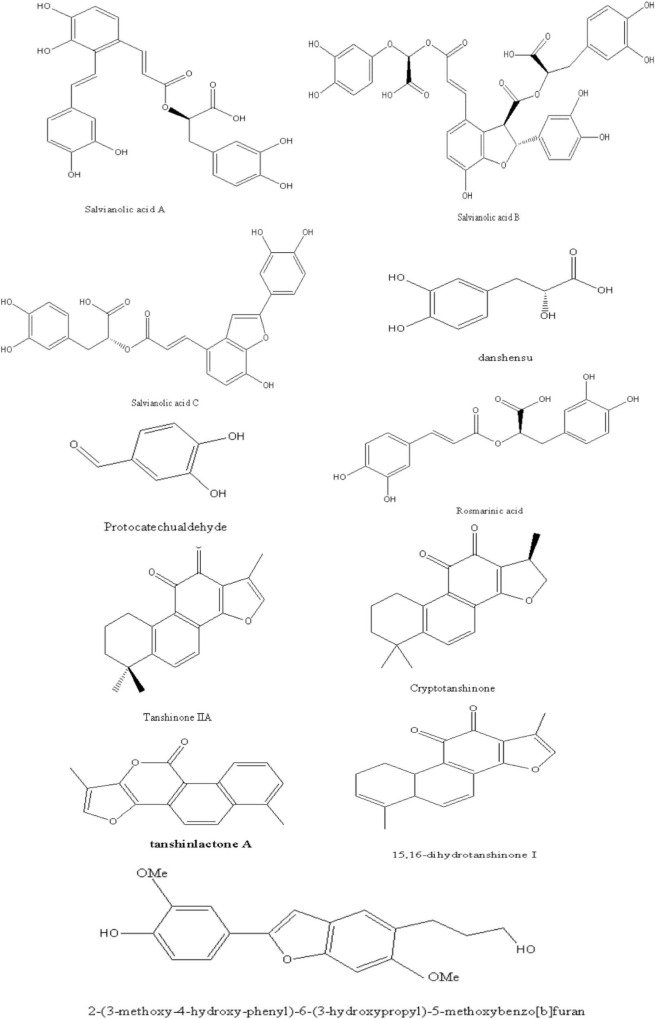
The compounds in Danshen related to the pharmacological effects of NAFLD.

In this article, we briefly presented the identified chemical structure in Danshen, which related to the pharmacological effects of NAFLD as Figure 1. Interested readers are inspired to know more compounds of Danshen refer to related reviews.

## Pharmacokinetics Characteristics of Danshen

Pharmacokinetics is a bridge linking herbal medicine and pharmacological responses. The active ingredients of Danshen exert therapeutic effects of NAFLD through entering the systemic circulation to reach the target organ or tissue. Painstaking efforts directed at mechanisms influencing the pharmacokinetics of Danshen can aid in improving their intended pharmacological activity. The major topics cover (i) Danshen ability to enter a system and how to metabolite and eliminate through various tissues, and (ii) what factors determine the course of Danshen bioavailability and how to improve it.

## Phenolic Acids

Extensive research performed the complicated absorption mechanism of phenolic acids of Danshen. The gastrointestinal absorption of rosmarinic acid (RA), salvianolic acid B (SAB), protocatechuic aldehyde (PCA) and TSL were characterized by passive diffusion in the intestine (24, 25). Besides, the paracellular absorption transport pathway also existed in SAB and TSL (26). The phenolic acids were of low bioavailability in animal or human plasma after oral administration, except for TSL. For instance, the oral bioavailability of SAB, salvianolic acid C (SAC), lithospermic acid (LA), salvianolic acid D (SAD), magnesium lithospermate B and miltirone were 1.07, 0.29, 1.15, 4.16, 0.02, and 3.4% in rats, respectively (27–31). Moreover, the concentration of Danshen preparations in plasma after oral administration slightly detectable in human subjects. Li et al. found that the C_max_ of TSL, PCA and protocatechuic acid (PAA) in humans were 134.1 ± 48.4, 10.5 ± 5.6, and 59.0 ± 18.6 ng/mL after oral administration of Compound Danshen Dripping Pills (32). Researches showed that the poor bioavailability of phenolic acids was due to their adverse properties, including molecular mass, hydrogen bonding capacity and molecular flexibility (33). TSL has good properties and its bioavailability reaches to 30–40% in animals (34). Injection is more effective than oral administration. The TSL, SAD, LA, RA, PCA, and TSL exhibited considerable exposure in human subjects and rats after intravenous administration Danshen (35, 36).

Polyphenol compounds were transformed by colonic microflora and decomposed into simple phenolic acid derivatives, easily absorbed from the intestine. The major metabolites of phenolic acids were the methylated and their sulfated and glucuronide products in humans, rats and dogs, which may be responsible for the biological activity (37, 38). SAA, SAB, PCA, and LA were subject to extensive elimination mainly via hepatobiliary excretion of glucuronide and renal excretion (37). For example, SAB seemed to enter liver cells rapidly after oral or intravenous dosing, and methylated metabolites may exert antioxidant action. PCA was transformed into PAA in the liver and then eliminated by conversion into sulfates and methylated glucuronides followed by renal excretion (36). However, RA, TSL, and SAD were eliminated mainly via renal excretion, rather than hepatobiliary excretion (34, 39). Among these, RA could be broken down into smaller phenolic acids such as TSL and caffeic acid and eliminated by renal excretion in healthy human subjects (40). Further studies with the metabolic pathways of other component in Danshen preparations are required.

Several presystemic processes, including gastrointestinal solubility, membrane permeability, gastrointestinal degradation, transporter-mediated intestinal efflux, systemic intestinal wall metabolism, and hepatic metabolism, could lead to the low bioavailability of phenolic acids. Researchers have been trying to improve the bioavailability of Danshen such as ameliorating dosage form (e.g., sodium caprate and lipid nanoparticle formulations). For instance, the sodium caprate could significantly enhance the bioavailability of both TSL and SAB by improving intestinal permeability (26). When encapsulated into liposomes and chitosan nanoparticles, the t_1/2_ and AUC_0–∞_ of SAB in beagle dogs were higher than that of free SAB (41, 42). Certain components in Danshen preparations can affect the pharmacokinetic behavior of other coexisting ingredients, which contributed to synergistic or metabolic transformation. Chang et al. observed that the AUC_0–8_, CL and t_1/2_ of TSL and SAB were significantly influenced by the content variation of the other major components in the Danshen injection (43). Moreover, the tanshinones affected the bioavailability and distribution of RA, SAB, and TSL, and accelerated the biotransformation of Sal B to TSL. Similarly, the phenolic acids could influence the biotransformation of CT to TSIIA and improve the bioavailability of TSIIA (44). Additionally, the common Danshen compound compatibility herbs such as borneol can also improve the bioavailability of Danshen (45). Therefore, it will be interesting to focus on the transformation or interaction of components between Danshen preparation in the future.

## Tanshinones

Few studies have reported the absorption mechanism of tanshinones. Yan et al. elucidated that the transport mechanisms of TSIIA and CT were active transport or facilitated diffusion (46). Zhang et al. observed that CT was transported primarily via an active mechanism across the intestinal epithelium, a saturable process that could pump CT into the luminal side (47). Meanwhile, other studies reported that TSIIA might not exclude a passive transport process (48, 49) due to its low molecular weight (294 D) and high lipophilicity (logP = 6.1) (50). The plasma level of tanshinones is low, generally in the nM to the sub-μM range in animals after oral or injection. Here offers instances that, Yu et al. found that the oral bioavailability of tanshinone IIB (TSIIB) was about 3% in rats (51), and the C_max_ of 0.274 μg/mL of TSI after intravenous injection at 3 mg/kg dose in rats (52). Additionally, the blood concentration of Danshen mixture was poor in rats, either. The C_max_ of TSI, TI, TSIIA, and CT were 1.63 ± 0.78, 3.23 ± 1.40, 2.78 ± 0.96, and 0.66 ± 0.27 ng/mL after oral administration of PF2401-SF (the standardized fraction of Danshen, equivalent to TSI: TI: TSIIA: CT = 1.15:1.10:4.1:1.91 mg/kg) to rats (53). When a mixture of phenolic acids and tanshinones including TSL (10.25 mg/kg), RA (6.39 mg/kg), CT (9.82 mg/kg), TI (13.58 mg/kg), TSI (3.90 mg/kg), and TSIIA (5.79 mg/kg) were orally administered to rats, the C_max_ of each constituent was 72, 37, 43, 11, 55, and 22 ng/ml, respectively (54). Exposure to tanshinones of granular powder formulation after a single oral administration in healthy volunteers, the C_max_ of TSI, TSIIA, and CT was 6.57, 25.8, and 146.7 ng/mL, respectively (55). The low bioavailability of tanshinone is mainly due to the lack of molecules with high logP or optimal HLB values and permeability. Therefore, bioavailability enhancement improved the application of tanshinones in the clinic.

Previous investigation indicated that the concentration of tanshinones in bile was much higher than in plasma and urine, suggesting that the tanshinones are mainly metabolized in the liver (56). TSIIA is preferentially distributed into the liver after either intravenous or oral doses, which is due to the self-association or self-assembly of highly hydrophobic compound to form macromolecules or polymers and can be recognized and taken up by the reticuloendothelial system (48). TSIIA underwent extensive CYP-mediated oxidation, and then subject to glucuronidation, after the glucuronides hydrolysis, aglycones are reabsorbed from the intestine and excreted into bile as a conjugate (57). TI was initially biotransformed into TSI and then shared the metabolic pathways, including glucuronidation, hydroxylation, reduction, methylation, and the O-sulfate conjugated reaction (58, 59). Hence, methylation, demethylation, dehydrogenation, hydrogenation, and hydroxylation were the major metabolic transformation of tanshinones.

The factors including poor solubility, poor permeability, and formulation, etc. resulting in low bioavailability of tanshinones. Similar to phenolic acids, the ultrafine powder and solid lipid nanoparticles formulations could improve the bioavailability of CT, TSIIA, TI, and TSI by enhancing their stability and permeability (60–62). On the other hands, components in tanshinone extracts also affected the pharmacokinetics of monomer constituents. Song et al. reported that tanshinone extracts synergically promoted the absorption of TSIIA and CT and accelerated the transformation of CT to TSIIA (63). The mechanism of promoting absorption and biotransformation between components in TCM formula should be further studied.

## The Polysaccharides of Danshen

In addition to phenolic acids and tanshinones, polysaccharides of Danshen exhibited antioxidant, immunoregulatory, ameliorating metabolic disease, hepato-protective, and against NAFLD (64). Few studies involved pharmacokinetics of Danshen polysaccharides compared with the pharmacodynamics. This is related to the complex molecular structure, which lacks both chromogenic and light absorption groups, and susceptible to the interference of biological substances in concentration detection. What’s worse, it is well known that polysaccharides are poorly absorbed. Generally, there are three theories for the mechanism of oral absorption of polysaccharides: direct absorption, intestinal microflora absorption and intestinal Peyer collection lymph node absorption (65). Wang et al. concluded that ganoderma lucidum polysaccharide was ingested by pinocytosis in Caco-2 cells (66). And the elimination of polysaccharides is mostly by the kidney (67). Some researchers believe that polysaccharides work through direct absorption in the gut. A novel polysaccharide of Danshen, SMWP-U&E, improved the absorption of weaned piglets by increasing the diversity and evenness of the intestinal microflora after doses at 1.5 g/kg to the pigs (68). Lack of evidence regarding the mechanism of the pharmacokinetics of polysaccharides is a major barrier to realizing the full potential pharmacological activity.

## Discuss the Activity Against Non-Alcoholic Fatty Liver Disease of Danshen Compounds With Low Bioavailability

The phenolic acids, tanshinones, and polysaccharides isolated from Danshen exhibited various biological activity such as against NAFLD. However, how some Danshen compounds with low bioavailability exhibit their pharmacological activity against NAFLD. Here we attempt to interpret this from four aspects: (i) the chemical structure-activity of Danshen, (ii) the bioavailability of Danshen compounds in comparison between physiological and physiological condition, (iii) pharmacological activity of the improved bioavailability compound of Danshen, (iiii) Danshen induced gut microbiota alteration.

## The Chemical Structure-Activity of Danshen

The preventive and therapeutic potential of Danshen on NAFLD is related to reducing the risk of metabolic disorders, including antioxidant, anti-inflammatory, immunoregulatory, etc. Normally, the chemical structures of phenolic acids influence their redox potential. Specially, polyphenols with two o-hydroxyl groups on an aromatic residue are more capable of scavenging free radicals than polyphenols with only one hydroxyl group (69). The other active constituents of Danshen, diterpenoids, were demonstrated to exert anti-inflammatory, immunosuppressive and antitumor activity. Quinone group and aromatic ring are the basic structure of tanshinone activity, and carbonyl may play an important role in its antitumor activity (37). Zheng et al. confirmed that the antiproliferative effects of abietane quinone diterpenoids against five cancer cell lines (70). On the other hand, in addition to the parent compounds, the metabolites usually exhibited pharmacological activity. For instance, the sulfate esters and glucuronides metabolites of phenolic acids were shown to retain part of their antioxidant properties (71). Therefore, many of the biological effects observed in animal or clinical studies may be explained by the microbial metabolites of those compounds. Much research effort is needed to evaluate the biological effects of the conjugated derivatives and microbial metabolites of polyphenolic acids and diterpenoids. Generally, the structure-activity relationship between polysaccharides and intestinal flora from molecular weight, glycosidic bond, and monosaccharide composition (72). However, Danshen polysaccharide has not been studied in this respect.

## The Bioavailability of Danshen Compounds in Comparison Between Physiological and Physiological Condition

The pharmacokinetics of Danshen formulation varies with pathological and physiological conditions due to the biological environment in the body. Accumulative studies have shown that the bioavailability of Danshen increased under pathological conditions compared to normal conditions. Shi et al. found that the plasma concentrations of TSL, PCA, PAL, SAC, SAB, and TSIIA and the AUC_0–t_, MRT_0–t,_ and t_1/2_ of SAB and TSL in the middle cerebral artery occlusion rats were significantly increased compared with the sham-operated group (73). The CL of STS was lower in coronary heart disease patients than in healthy volunteers in a population pharmacokinetic model study (74). Moreover, several metabolites of TSIIA presented differences in the distribution between the sham control and the Alzherimer’s Disease Rat model (75). The pharmacokinetics of Danshen in the NAFLD disease state needs to be studied since several studies have demonstrated the altered pharmacokinetics of drugs in NASH patients (76).

## Pharmacological Activity of the Improved Bioavailability Danshen Compound

The modified Danshen compounds improved bioavailability and pharmacological activity. Li et al. reported that STS was a promising drug for treating NAFLD by decreasing lipid accumulation and suppressing inflammation (77). Moreover, TSIIA encapsulated into globin to form nanoparticles that could markedly attenuate the progression of hepatic fibrosis (78). The nanoparticle-containing TSIIA was significantly more effective in inhibiting tumor growth in mice with hepatoma than free TSIIA (79). The pharmacological activity of modified other compounds from Danshen need further study.

## Danshen Induced Gut Microbiota Alteration

Hypothesized mechanisms of how the gut microbiome contributes to the development of NAFLD by increasing intestinal permeability, leading to the release of lipopolysaccharide into the host, which can trigger tissue and systemic inflammation, and metabolite produced by microorganisms that can influence immunity (80, 81). Currently, increasing evidences showed that Danshen preparations and the monomer active components regulated intestinal flora. For example, the aerial parts of Danshen could imbalance the intestinal microflora disorder caused by diabetes (82). Wang et al. observed that SAA modulated gut microbiota imbalance during colitis by increasing the gut microbial diversity as well as selectively promoting some probiotic populations (83). Many preparations containing Danshen balanced the intestinal flora such as SMWP -U&E (68), BuZangTongLuo decoction (84), the mixture of yeyachun and Danshen (85). Among these, Danshensu Bingpian Zhi not only reversed HFD-induced intestinal microbiota dysbiosis but also stimulated brown adipose tissue browning and maintains intestinal barrier integrity (86). Previous studies indicated that microbiota composition is related to insulin resistance (87, 88). Probiotics and Danshen polysaccharide combination has the potential to be used as a therapeutic for ameliorating NAFLD via regrouping the composition of the intestinal microbiota and improvement of insulin resistance. In addition, Bacteroidetes/Firmicutes ratio can be increased by Danshen polysaccharide, which is a compositional biomarker for obesity and type 1 diabetes mellitus (89). Danshen preparation including Jian-Gan-Xiao-Zhi (90), DLT-SM (91), and GuanXinNing Tablet Decoction (92) decreased the Firmicutes/Bacteroidetes ratio through regulation of host metabolism. Recent study has found the genera Muribaculaceae might be concerned with the resistance effect of lean mice to HFD (93). Meanwhile, the reduction of Erysipelotrichaceae may be beneficial to lipid metabolism (94). Dingxin Recipe IV increased the relative abundance of Muribaculaceae and decreased Erysipelotrichaceae, which is beneficial to lipid metabolism (95). However, the mechanism of those bacteria in the development of lipid metabolism remains unclear. Besides, there is an intense correlation between fecal microbiota and inflammatory factors. For example, TI enriched bacterial species which promote butyric acid metabolism or negatively correlated with inflammatory factors (89). Interestingly, the effect of water extract on root and rhizome of Danshen was stronger than that of alcohol extract, which further confirmed that Danshen could improve intestinal microflora disorder (96). Most evidence in this field comes from animal experiments and further human study is needed.

## Danshen in Management of Disease Based on Metabolic Targets Related With Non-Alcoholic Fatty Liver Disease

### Mechanisms of Metabolic Targets Regulating the Progression of Non-alcoholic Fatty Liver Disease

**FIGURE 2:**
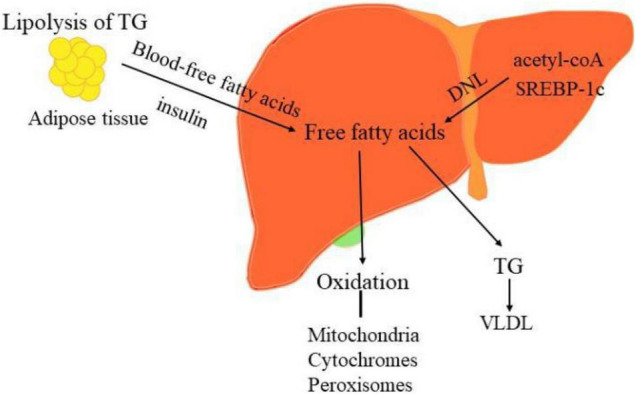
The sources and disposal of hepatic free fatty acids. The key pathogenesis of NASH are hepatic free fatty acids. The liver acquires free fatty acids through DNL and lipolysis of triglyceride in adipocytes. Phosphorylation of JNKs in adipose tissue dramatically impacts insulin signaling, which contributes to excessive delivery of lipids to the liver and leads to NASH. Moreover, increased DNL also result in the elevated liver lipid content in NAFLD. DNL can be pharmacologically inhibited by targeting its synthetic enzymes acetyl-CoA, SREBP-1c, and ChREBP. Conversely, the disposal of fatty acids including oxidation in the mitochondria, cytochromes, and peroxisomes and formation of triglyceride (TG).

From the theory of “second two-hit” to “multiple hits,” the pathogenesis of NAFLD only partially understood so far. When determining the pathogenicity drivers of NAFL and NASH, elucidating the source and fate of fatty acids in hepatocytes is vital to interpret the metabolic basis of NAFLD. The liver acquires free fatty acids through *de novo* lipogenesis (DNL) and triglyceride lipolysis in adipocytes, as shown in Figure 2. Primarily, fatty acids are delivered from the blood to the liver through the lipolysis of triglycerides in adipose tissue, regulated by the insulin on adipocytes. Phosphorylation of JNKs in adipocytes dramatically impacts insulin signaling, which contributes to excessive delivery of lipids to the liver and leads to NASH. Secondly, the increased peripheral fatty acids and DNL also elevated liver lipid content in NAFLD. DNL can be pharmacologically inhibited by targeting synthetic enzymes, such as acetyl-CoA, SREBP-1c, ChREBP (12, 97).

**FIGURE 3:**
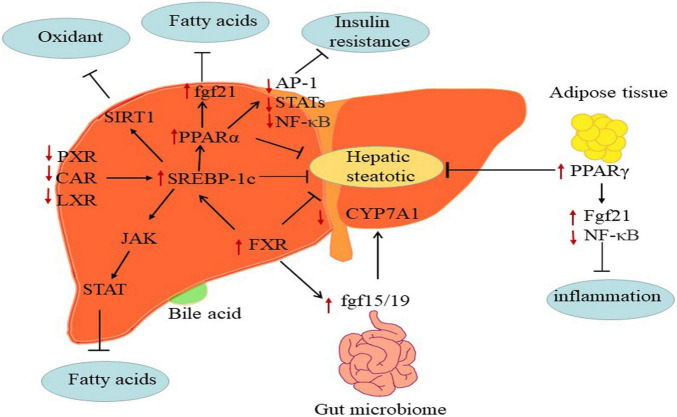
The mechanisms of major metabolic targets regulating the progression of NAFLD. PPARs: The FGF21 directly targeting PPARs key pathogenesis of NASH are hepatic free fatty acids. And activated PPARα may negatively interfere with the activity of pro-inflammatory transcription factors AP-1, STATs, and NF-κB. PPARγ attenuates inflammation via inhibiting NF-κB activity and elevating FGF21 in adipose tissue. FXR: FXR regulates hepatic bile salt synthesis through stimulating FGF15 (FGF19 in humans) expression in the intestinal and repressing CYP7A1 in the liver. LXR: Inhibition of LXRα via SREBP-1c contributes to alleviate progress of hepatic steatosis. PXR and CAR: PXR and CAR regulates SREBP1 involved in metabolic homeostasis.

Besides, nuclear receptors including PPARs, retinoid X receptorα (RXRα), liver-X-receptorα (LXRα), farnesoid X receptor (FXR), pregnane X receptor (PXR), and constitutive androstane receptor (CAR) regulated fatty acids (Figure 3) (98). Moreover, NAFLD is poised to have an appreciable impact on the expression and function of ATP-binding cassette efflux transport proteins (e.g., MRPs), uptake transporters (e.g., OATPs), and metabolic enzymes such as CYP, UDP-glucuronosyltransferases (UGT) and sulfotransferases (SULT) (99–101).

The PPARα, PPARβ/δ, and PPARγ are members of the nuclear receptor PPAR super-family. PPARα, which is expressed at the high levels in the liver. The deletion of this gene gradually as NASH progresses accelerated the development of NAFLD in preclinic and clinic (102). The activation of PPARβ/δ regulated hepatic glycolipid metabolism in NAFLD (103). PPARγ is mainly in adipose tissues, where its ligands enhanced adipocyte storage of fat and contributed to improve NASH (104). In short, PPARs exerted functions in glycose and lipid metabolism, inflammation, and fibrosis by modulating the expression of specific target genes (105). Indeed, the fibroblast growth factor 21 (FGF21) is an effective metabolic modulator of hepatic glycolipid homeostasis and insulin sensitivity, and PPARs directly induces FGF21 expression in the rodent liver (106, 107). Besides, activated PPARs may negatively interfere with the activity of pro-inflammatory transcription factors such as activator protein-1 (AP-1), signal transducers and activators of transcription (STATs), and nuclear factor-κB (NF-κB) and therefore repressing fibrosis progression (108–110). For instance, PPARα-agonists, fibrates, have shown vital benefit in treating cardiovascular disease in clinical (111) and NAFLD in rodents (112). However, the efficacy of fibrates on NAFLD treatment in humans has not been clarified. Moreover, PPARs modulated lipid dysregulation and inflammatory by adiponectin. Overexpression of PPARγ detected in NAFLD, which is potentially due to adiponectin (113, 114). Researches showed that the agonists of PPARγ against NAFLD might attributed to induce adiponectin (115, 116).

FXR, the bile acid receptor, negatively modulated bile acid synthesis and reduced hepatic gluconeogenesis, lipogenesis and steatosis at both hepatic and extrahepatic tissues (117). The hepatic expression of FXR downregulated in NAFLD patients. Activated FXR appears to influence insulin sensitivity and lipoprotein transport at multiple levels, which indicated that FXR holds promise as targeted therapeutic (118). FXR regulated hepatic bile salt synthesis by stimulating FGF15 (a hormone with direct glycogen synthesis in the liver and distant organs, FGF19 in humans) expression in the intestinal and repressing CYP7A1 in the liver (119, 120). Activated FXR improved hepatic glycogen synthesis via inducing FGF15 in mice (121). Of note, FXR agonist CDCA affected glucose homeostasis by controlling the expression of glucose transporter 4 (GLUT-4) (122). Meanwhile, activation of FXR also suppressed the formation of hepatic lipid and stimulated FAβ-oxidation to limit lipid accumulation. At present, BA-derivative OCA (FXR agonists) has been studied in patients with NAFLD, and further studies are needed to better define the clinical usefulness of OCA in NASH (123).

LXR regulated liver cholesterol homeostasis, inflammation, and fibrosis *in vitro* and in patients with NAFLD (124, 125). LXRα is responsible for lipid metabolism mainly in the liver, whereas LXRβ expressed ubiquitously (126). Higuchi et al. observed that the expression of LXRαincreased in patients with NAFLD (127). Morin, a dual antagonist of LXRα and LXRβ, alleviated hepatic steatosis and metabolic disorders via the suppression of LXR signaling (128). Namely, LXR antagonism may be productive for attenuating hepatic steatosis and ensuing fibrosis.

PXR and CAR are xenobiotic-sensing nuclear receptors that modulated the expression of genes such as CYPs, UGTs, and OATPs (129). More recently, PXR and CAR participated in regulating glucose, lipid, and bile acid metabolism, and highly expressed in the liver and gut (130). Previous studies have shown that activated PXR and CAR worsen the hepatic steatosis and insulin resistance in NAFLD by suppressing gluconeogenesis and β-oxidation and increasing hepatic fatty acids uptake (131, 132). Indeed, PXR also regulated SREBP1 and PPARα involved in metabolic homeostasis (133, 134), and CAR targeted CYP2B6 (99). For example, a study demonstrated that SREBP-1 inhibited drug-mediated induction of CYP2B and CYP3A via activating PXR and CAR in rodents, which indicated that PXR and CAR respond to lipid accumulation through direct interaction with SREBP-1 (135).

Due to its progressive nature and its significant impacts on hepatic histopathology, NAFLD is expected to significantly affect transporters and metabolic enzymes such as CYPs, UGTs, and SULTs. The expression and function of transporters such as OATPs and MRP changed with NAFLD progressing in humans and animals. This change affected the plasma and tissue disposition of endogenous and exogenous compounds (136). It seems to be that the CYP2E1 and CYP3A have been identified as the most relevant enzyme due to the vital role of oxidative stress in NAFLD (137). The induction of CYP2E1 is an adaptive response, which prevented lipid overload in NAFLD. While the increased CYP2E1 and improved insulin resistance appear to stimulate each other, that may ultimately worsen the process of steatosis with the increase in oxidative stress (138). However, the mechanism is not clarified yet. CYP3A genes appear to be regulated by various signaling pathways such as CCAAT-enhancer-binding proteins (C/EBP), HNF, PXR, and CAR, Janus kinase/signal transducers and activators of transcription (JAK/STAT), and mitogen-activated protein kinases (MAPK) pathway (19). However, few study have been reported that elucidating the shifts in the drug metabolism and bile acid associated with UGTs and SULTs enzymes in NAFLD to date. Hardwick et al. showed minimal alterations in the activity of UGTs while several changes in the expression and function of specific SULT during human NAFLD progression (101). The bile acid-related enzymes UGT and SULT2A1 were strongly suppressed in high-fat-cholesterol-fed males (139, 140). Expression and functional of transporters changed in NASH patients. OATP1B1 and OATP1B3 downregulated, MRP3 and MRP4 upregulated and MRP2 mislocalized in NAFLD (141). The mechanism is that the loss of glycosylation of the transporters (142).

### The Pharmacological Activity of Danshen in the Management of Non-alcoholic Fatty Liver Disease

Concerning the therapy for NAFLD, we focus mostly on risk factors such as metabolic syndrome, including overweight, hyperglycemia, insulin resistance, dyslipidemia, and hypertension (143). Conversely, the NAFLD may strengthen some features and comorbidities of the metabolic syndrome. Especially, cardiovascular diseases are the main causes of mortality of the patients with NAFLD (5). Thus, effective treatment of NAFLD and metabolic syndrome could have mutual benefits for each other. Danshen is a highly versatile and multi-activity herb that can significantly limit the processes of liver diseases. Clinical trials of Danshen effectively treat NAFLD and metabolic syndrome (9, 10, 144). More evidence indicated that Danshen and their derived compounds limited the progression of hepatic steatosis of NASH in animals and *in vitro* (145–147). For example, Danshen tablets combined with Alovastatin significantly alleviated fatty liver cirrhosis and cardiovascular atherosclerosis caused by abnormal lipid metabolism in NAFLD treatment (148). Danshen polysaccharides combined with Probiotics regulated lipid metabolism disorders and protected the liver in NAFLD mice (8). Conclusively, the preventive and therapeutic potential of Danshen for NAFLD is related to reducing the risk of metabolic disorders.

**FIGURE 4:**
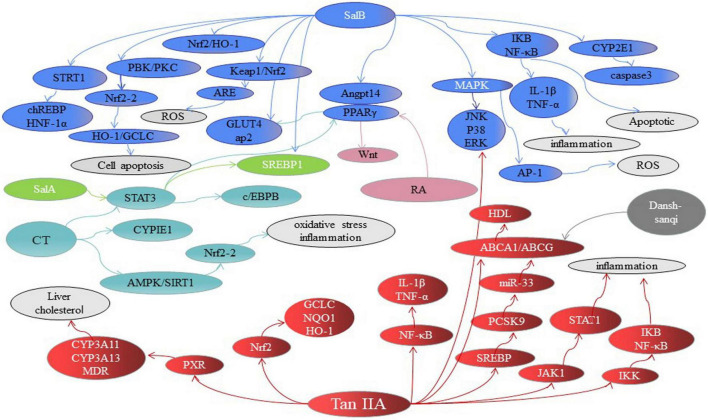
The major signaling pathways involved in treating NAFLD of Danshen compounds. SAB, Salvianolic acid B; SAA, Salvianolic acid A; SAC, Salvianolic acid C; RA, Rosmarinic acid; TSIIA, tanshinone IIA; CT, Cryptotanshinone. FA, fatty acids.

A variety of mechanisms in inhibiting hepatic steatosis and modulating lipid metabolism have been shown by the compounds of Danshen and its preparations (Table 1), including the regulation of hepatocyte apoptosis via inducing autophagy in an AMPK-dependent way (149), the modulation of lipid metabolism via activating ChREBP (146), PPARα (7), PXR (150) or inhibiting PPARγ (151) and LXR (95), anti-inflammation through inhibiting Nrf2 (152) and JNK (153), anti-oxidative stress by activating Nrf2 and suppressing CYP2E1 (154), anti-fibrosis through inhibiting MAPK (155), regulating NF-κB/IκBα (156), activating Wnt/β-catenin (157) and SIRT1/HSF1 (158) and improving the insulin resistance (159). For instance, SAB against hepatocyte apoptosis by upregulating the mortalin, a protein of maintaining mitochondrial homeostasis (160). CT exhibits a hepatoprotective effect by activating Nrf2 and AMPK/SIRT1 and inhibiting CYP2E1 (161). SAB could effectively inhibit liver fibrosis with 60 patients (162). Potentially, the combination of Danshen Polysaccharide and Probiotics ameliorated NAFLD via controlling the gut microbiota and insulin resistance (8). In particular, the genes including JNKs, SREBP-1c, ChREBP, PPARs, CYPs, and the others have been highlighted as crucial molecular targets for Danshen treating patients with NAFLD (Figure 4).

**TABLE 1:** The metabolic targets involved in treating NAFLD of Danshen.

Herb	Animal/cell model	Dose	Targets/pathways/mechanisms
CT	HepG2 and MEF, A549, DU145, AGS, and HCT116 cells	5–10 mM	AMPK
Danshen	CDAA diet-induced mice	0.093–0.84 g/kg	PPARα/JNK
TSIIA	Lipopolysaccharide-induced hepatic stellate cells	1–10 mM	JNK and JAK/STAT
TSIIA	Lipopolysaccharide-induced ATDC5 cells	5–20 μM	JNK and JAK/STAT
CT	Sodium-nitroprusside-induced neuro-2a cells	10–20 μM	ERK1/2 and JNK
Tanshinlactone A	Human peripheral blood mononuclear cells	6.25–100 M	ERK, p38, and JNK
TSIIA	Lipopolysaccharide-induced-RAW 264.7 cells	1–10μg/mL	NF-κB, p38, ERK1/2, and JNK
SAC	APAP-induced mice	5–20 mg/kg	AP-1and JNK
15,16-dihydrotanshinone I	U2-OS, HEK293T, HK-2, and MIN6 cells	2.5–20 μM	Acetyl-CoA, AMPK
SAB	db/db mice	50-100 mg/kg	Acetyl-CoA and PPARα of AMPK
TSIIA	Hyperlipidemic rats	10 mg/kg	miR-33a and SREBP2/Pcsk9
Danshen-Sanqi preparation	High calorie food induced mice models	0.4–0.8 g/kg	GLUT-1, GK, GLUT-4, and SREBP-1c
Dingxin Recipe	ApoE-/- mice	1.8–0.45 g/kg	LXRα/SREBP1
TSIIA	Lithocholic acid- induced mice	5–20 mg/kg	PXR, Cyp3a11, Cyp3a13, and Mdr1
SAA	High-fat diet- induced rats	8–16 mg/kg	ChREBP and TXNIP
SAB	Ethanol induced rats	15–30 mg/kg	SIRT1/CRP and ChREBP
Danhong injection	High-fat diet rats	1–2 mL/kg	acetyl-CoA and PPAR-α
Danshen injection	Alcohol-fed rats	3 g/kg	PPARα and 4-Hydroxynonenal
TSIIA/standardized fraction of Danshen	Lipopolysaccharide-induced RAW 264.7 cells	1–50 μM 1–50 μg/mL	RXRα
SAA	Prednisone in adriamycin-induced rats	10 mg/kg	Nrf2/HO-1 and PPARγ/Angptl4
SAA	Prednisone in adriamycin-induced MPC5 cells	50 μmol/l	PPARγ/Angptl4
RA	Ligation and scission of the common bile duct- induce mice	0.1 mg/25 g	Wnts/PPARγ
TSIIA	Preadipocyte3t3-L1 cells high-fat diet induced mice	5–50 μM	PPARγ
TSIIA	Human adipose fibroblast cells	0.1–30 μM	PPARγ
SAB	High-fat diet mice	100 mg/kg	PPARγ, C/EBPα, GATA-2 and GATA-3
SAB	High-fat diet mice	100 mg/kg	PPARγ/SREBP-1, c/EBPα
CT	Preadipocyte3t3-L1 cells	2–10 μM	PPARγ/STAT3
Preparation Danshensu Bingpian Zhi	High-fat diet mice	50–100 mg/kg	PPARγ
2-(3-methoxy-4-hydroxy-phenyl)-6-(3-hydroxypropyl)-5-methoxybenzo[b]furan	Preadipocyte3t3-L1 cells	1–25 μM	PPARγ, C/EBPα
PCA	Human adipose fibroblast cells	10–100 μM	PPARγ, C/EBPα, C/EBPβ
SAB	Preadipocyte3t3-L1 cells	50 μM	PPARγ, PPARα, C/EBPα
Danshen extract	Carbon tetrachloride-induced rats	10–100 mg/kg	CYP2E1
CT	Ethanol- induced mice	20–40 mg/kg	AMPK/SIRT1, CYP2E1
Polysaccharides of Danshen	CCL_4_-induced primary hepatocytes	100 μg/kg	ALT, AST, malondialdehyde, GSH, and CYP450
TSIIA	Acetaminophen-induced mice	10–30 mg/kg	Nrf2/GCLC, HO-1

## Possible Mechanisms of Danshen in Management of Non-Alcoholic Fatty Liver Disease Based on Metabolic Targets

### Fatty Acids in the Non-alcoholic Fatty Liver Disease Control the Activity or Expression of Key Metabolic Targets

#### c-Jun N-terminal kinases

JNK is a critical mediator of insulin resistance, which leads to NASH through dysregulated lipolysis causing excessive transmit of fatty acids to the liver and intracellular accumulation of toxic lipid products that impair insulin signaling and activate inflammatory pathways (163). Several compounds of Danshen exhibits hepatoprotection and anti-inflammatory and subsequently against NAFLD through JNK-related signaling pathways. TSIIA, TSL, and SAB alleviated NAFLD progression through targeting PPARα and PPARα/JNK signaling pathways. TSIIA inhibited phosphorylation of JNK on lipopolysaccharide-induced rat hepatic stellate cells, and TSIIA exhibited stronger effects of hepatoprotection than SAB (228). Similarly, CT effectively against apoptosis by blocking the activation of extracellular signal-regulated protein kinase (ERK)1/2, NF-κB and JNK signaling pathways (165). Additionally, Tanshinlactone A, a new diterpenoid tanshinone compound from Danshen, which against inflammatory through inhibiting the ERK, p38 and JNK in cells (166). NF-κB regulated inflammation by phosphorylation of IκB via activation of the p38, ERK1/2 and JNK (167). TSIIA (168) and SAC (169) presented the anti-inflammatory activity through the signaling pathways mentioned above.

#### Sterol Regulatory Element-Binding Protein-1c

Free fatty acids are central to the pathogenesis of NASH. The main source of fatty acids from food or synthesized by acetyl-CoA through complex reactions including glycolysis and citric acid (170). AMPK is an energy mediator, which controlled cellular lipid metabolism. Phosphorylation of acetyl-CoA promoted adipogenesis by deactivating AMPK and inhibiting lipid oxidation (171). TI potently enhanced acetyl-CoA phosphorylation which caused decreased lipogenesis (172). Moreover, PPARα is one of the major downstream targets of AMPK (173). SAB relieved dyslipidemia and hyperglycemia partly by modulating the acetyl-CoA and AMPK/PPARα in db/db mice (174). Moreover, AMPK interacted with SREBP-1c and SREBP-2 and directly phosphorylated in diet-induced insulin-resistant mice (175). SREBP-1C, a major transcriptional regulator of the acetyl-CoA in the process of synthesis, could benefit the treatment of NASH (12). And signal transducer and activator of transcription (STAT-3) related to lipid synthesis by modulating the expression of SREBP-1C (176). Salvianolic acids significantly balanced the lipid metabolism disorders by inhibiting STAT-3 via suppressing the expression of SREBP1 (177). Meanwhile, SREBP-2 regulated the expression of Pcsk9 in hepatic cholesterol (178). A miRNA located in the SREBP-2 gene, microRNA (miR)-33, modulated lipid metabolism through regulation of ATP-binding cassette transporter A1 (ABCA1) and G1 (ABCG1) (179). TSIIA ameliorated lipid accumulation in the hyperlipidemic rats via modulating the miR-33a and SREBP2/Pcsk9 pathway without impacting the lipid profile serum (180). Besides, the combination of herbs usually exerted synergistic effects. Danshen-Sanqi prescription increased the expression of hepatic glycogen synthesis gene, GLUT-1, GK, and GLUT-4, and reduced SREBP-1c gene related to fat and cholesterol anabolism (181). Generally, downregulation of SREBP-1C expression is likely due to the modulate of FXR (12). Besides, LXRα, SREBP-1c and acetyl-CoA regulated fatty acid and cholesterol synthesis through the tricarboxylate transport system (182). For instance, Dingxin Recipe containing Danshen regulated lipid metabolism by LXRα/SREBP1 but not LXRβ and SREBP2 in ApoE-/- mice fed with a high-fat diet (95).

#### Carbohydrate Response Element–Binding Protein

Theoretically, ChREBP induced the expression of the acetyl-CoA involved in DNL in NAFLD conditions (183). SAA alleviated hepatic lipid accumulation of high-fat diet-induced NAFLD rats partially due to the cross-talk mechanism between ChREBP and Thioredoxin-interacting protein (TXNIP) (146). SAB played a critical role in antisteatotic and anti-inflammatory by activating SIRT1-mediated suppression of CRP (C-reactive protein, HNF-1α promoter) and ChREBP expression in rats (184). However, Benhamed et al. observed that ChREBP expression is increased in patients with NASH, 50% decreased in livers of severe insulin resistance patients, which suggested that ChREBP-mediated steatosis is not strongly associated with high insulin resistance (185), so it is uncertain whether ChREBP would be a viable target for therapeutics of NASH patients with severe insulin resistance.

#### Peroxisome Proliferator-Activated Receptors

Diversion of fatty acids away from liver into other tissues such as peripheral adipose tissue that reduced their transport to liver. PPARs have emerged as lipid sensors that transcriptionally modulated the metabolic process (104). PPARα and PPARγ are PPAR isotypes, which exhibited distinct functions.

PPARα is activated by fibrates and modulated the adaptive response to nutritional inputs by regulating fatty acid delivery in the liver. Furthermore, the mRNA level for PPARα progressively reduced as NASH progresses in humans (102). Hence, PPARα activation is central to the remission of hepatic steatosis and NAFLD progression. Recent studies demonstrated that the active constituents of Danshen and related preparations enhanced PPARα in the livers of rodents and thus reduced lipid accumulation. For instance, a pharmacological network analysis suggested that the mechanism of hepatoprotective effects of TSIIA, TSL, and SAB may involve modulating lipid metabolism and anti-fibrogenesis via PPARα, CYP1A2, and MMP2 (7). Besides, Danhong injection significantly enhanced lipolysis and diminished fatty acids’ synthesis in the liver by increasing the mRNA transcription of PPARα in hyperlipidemia rats (186). Of note, cellular protection is associated with activated PPARα involvement in 4-Hydroxynonenal metabolism (187). Ding et al. found that Danshen injection played a mechanistic role in hepatoprotection in mice via inducing the activation of PPARα and subsequent degradation of 4-Hydroxynonenal (188). Worth the whistle, 4-Hydroxynonenal degradation promoted by PPARα activation is likely to relate with multiple enzyme systems, which deserves further study. In addition, the action of RXRα as a heterodimer partner with PPARα promoted the expression of RXRα that mediated fatty acid transport to mitochondria and oxidation (189). Yin et al. found that TSIIA reversed lipopolysaccharide-induced the decreased gene expression of RXRα (190). Nevertheless, the proof of other ingredients of Danshen has not been brought yet.

In contrast to PPARα, the expression of PPARγ in the liver increased as steatosis develop in rodents. SAA combined with prednisone exhibited therapeutic and antiproteinuric effects on adriamycin-induced minimal change disease rat model through PPARγ/Angptl4 and Nrf2/Heme oxygenase-1 (HO-1) pathways (191). Further, PPARγ is considered as a downstream transcriptional target of Nrf2 in adipocytes differentiation (192). Hence, the influence of SAA modulated PPARγ/Angptl4 pathway is directly associated with Nrf2 could be studied in future. Wnt10b is a direct target of necdin, and necdin-Wnt pathway induced trans-differentiation of hepatic stellate cells through epigenetic inhibition of PPARγ (193). RA against liver fibrosis via inhibiting the expression and signaling of canonical Wnts/PPARγ in hepatic stellate cells (151). Similarly, it has been shown that SAB moderated lipid disorders by suppressing PPARγ-mediated adipogenesis in mice with high-fat diet-induced obesity (194). TSIIA inhibited adipogenesis as a natural antagonist of PPARγ in high-fat diet-induced obese mice (195). CT was demonstrated as an effectively anti-adipogenesis candidate through a multimodal signaling pathway- related to PPARγ (196). Moreover, PCA,2-(3-methoxy-4-hydroxy-phenyl)-6-(3-hydroxypropyl)-5-methoxybenzo[b]furan (an active compound identified from Danshen) and Danshensu Bingpian Zhi are efficient natural PPARγ agonists that exhibited excellent effects on insulin resistance, antiadipogenic, hepatic steatosis and inflammation (197, 198).

#### The Role of CYP450 Enzymes in the Pathogenesis of Non-alcoholic Fatty Liver Disease

Patients with NAFLD are more susceptible to drug- induced toxicity due to altered drug metabolism (19). Previous workers reviewed the change of human hepatic CYP450 enzymes in NAFLD, such as CYP2E1,3A4, 1A2, 2A6, 2B6, 2C8, 2C9, and 2C19 (199). Generally, it seems to be that the alter in CYP2E1 and 3A4 activity is predominant in clinic studies (19, 200). CYP2E1 is related to the regulation of oxidant stress, insulin resistance and fatty acids, and the expression of hepatic CYP2E1 enhanced in patients with NAFLD (201). Danshen aqueous extracts containing TSL and SAB protected hepatocytes from Paracetamol-induced injury via remaining mitochondrial metabolic activity and suppressing the activity of CYP2E1 and total glutathione depletion (202). As mentioned above, CT and SAC reduced the content of CYP2E1 (161, 169). Previous studies about the expression and function of CYP3A4 associated with NAFLD are a contradiction. Recently, the recent consensus is more in favor of the protein expressions and mRNA of CYP3A decreased in NAFLD (203, 204). CYP3A4 is in charge of the oxidative metabolism of over 50% of all drugs prescribed in NAFLD (203). CT and TSIIA activated PXR and subsequently induced CYP3A4 (205). CYP1A2 promoted the generation of ROS to facilitate oxidative damage further. And the active ingredients of Danshen, isoimperatorin and oleanolic acid attenuating oxidative stress by modulating the expression of CYP1A2, 2B6, and 1B1 in the liver (7). TSIIA protected against lithocholic acid-induced liver cholestasis due to the upregulation of PXR, as well as CYP3A11, CYP3A13, and MDR1 (150). Additionally, Danshen polysaccharides exhibited preventive success on the injury of chicken hepatocytes via reducing the contents of ALT, AST, and malondialdehyde and upregulating GSH and CYP450 (206).

#### The Role of the Other Proteins in the Pathogenesis of Non-alcoholic Fatty Liver Disease

Similar to CYPs, Phase II drug metabolic enzymes including UGTs, SULTs, and transporters (e.g., OATPs, MRPs) play various regulatory roles in NAFLD. UGT1A6, UGT1A9, and SULT1A1, SULT2A1 protein levels were downregulated in the liver of NASH patients (101, 139). Literature reports that the tanshinones are the inhibitors/substrates of UGT1A6 and UGT1A9 (207). However, whether Danshen treating NAFLD through UGTs and SULTs remains unclear. Besides, the induction of phase II detoxification enzymes HO-1 and glutamate-Lcysteine ligase catalytic subunit (GCLC) plays an antioxidant role in the Nrf2 pathway in NAFLD (208). TSIIA alleviated acetaminophen-induced liver injury by activating Nrf2 and its target genes such as GCLC and HO-1 (209). Additionally, the expression and function of OATPs changed with the disease progression of NAFLD, and LA, RA, SAA, SAB, SAD, and TSL are potent inhibitors of OATP1 and OATP3 (210, 211). It is worth mentioning that the other metabolizing enzymes and transporters of drugs altered in NASH patients when compared with healthy human livers, and the change deserves to explore to prevent adverse drug reactions.

#### Clinic Studies

Clinical trials have shown that Danshen improved NAFLD therapy. Danshen prescriptions such as Danshen powder injection, Danning tablet and Sangming Mixture are effective in patients with NAFLD by improving the symptoms, liver function and blood lipids with no serious side effects (10, 212, 213, 226). Hu et al. observed that Yindan Xinnaotong Soft Capsule is effective in the treatment of NAFLD by regulating lipid and insulin resistance and liver function and anti-inflammatory (214). Hence, the herbal formula that have proved to take positive effects on the physiological features of NAFLD. Clinically, the combination therapy of Danshen and other agents including probiotics (e.g., *Bacillus subtilis* enterococcus dual living bacteria capsule) (164), hepatic protectants (e.g., Magnesium isoglycyrrhizinate, Reduced glutathione, Tiopronin) (215–217), statins (e.g., Alvastatin) (148), and even the apparatus with liver disease treatment (227) has been already practiced, which presented better beneficial effects than interference alone. For instance, compound Danshen dripping pill combined with polyene phosphatidyl choline exhibited more effective in patients with NAFLD (218). Although claimed to be hepatoprotective in NAFLD, the molecular mechanisms of combination therapy with Danshen have not been clarified, further multicenter large-sample randomized clinical trials are required to confirm the therapeutic and safety.

SAB regulated insulin resistance mainly through the AMPK/GLUT4 and/or SREBP-1/PPARγ signaling pathway, and modulated fatty acids through PPAR-mediated pathways, and exerted the effect of anti-inflammatory by STRT1/chREBP. TSIIA exhibited the role of anti-inflammatory anti-oxidant by regulating signals such as MAPK, JAK/STAT, and PXR/CYP and modulated fatty acids through PPARγ and SREBP pathways. CT exerted the effect of anti-oxidant by CYP2E1 and regulated fatty acids through AMPK and PPAR-mediated pathways. SalA and SalC rebalance the lipid metabolism by PPARγ and CYP2E1, respectively. RA prevented liver fibrosis by PPARγ pathways. Each color represents a compound, and dotted line indicates inhibition effects.

## Future Prospects

Although steady progress has been made in elucidating the pathogenesis of NAFLD, determining therapeutic targets and advancing drug development, there are still unresolved challenges. To determine the molecular mechanisms, discern potential therapy targets, and guide the preclinic use of single or drug combinations, more studies are urgently needed using *in vitro* and animal NAFLD models. Many advances in the development of preclinic models such as *in vitro* models and genetic and dietary animal models for NAFLD have been made that have provided valuable insights on disease pathogenesis. However, only a few models resemble the key elements needed to be representative of NAFLD, while there exists a substantial difference between rodents and humans, such as the metabolic and transcriptomic characteristics, including the altered immune system and the altered glycolipid metabolism (219). Furthermore, no published data indicate that if drugs are effective in a given model, they consistently translate into efficacy in humans (220, 221). In addition, the heterogeneity of NAFLD according to sex, ethnicity and geographic region should be taken into consideration by researchers for clinic trials (222). Besides, the poor bioavailability, the most appropriate doses, the optimal route of administration, frequency of drug administration and the duration of treatment by Danshen are needed for various animal models to study comprehensively. Specifically, it is vital to focus on the experiment design, such as group size, controls and animal species.

Moreover, the tendency of this field is moving rapidly toward combination therapies, likely due to concern about insufficient ability to attack a single target, as shown that combining herbal medicine with other interventions presented better beneficial effects than interference alone (223). Besides, it is expected that researchers should discover more potent interplayed targets to increase synergy effects in future, such as PPARα agonist + PPARγ agonist, FXR agonist + PPARγ agonist, and PPAR pan-agonist + LXR antagonist (224).

Finally, the verification of predictive biomarkers of disease risk is lacking and urgently needed. Importantly, genetic risk factors such as the PNPLA3 gene have been identified that increase the NASH risk (225).

## Author Contributions

JL, YS, DP, NY, GW, LW, and WC wrote and contributed to the writing of the manuscript. All authors contributed to the article and approved the submitted version.

## Conflict of Interest

The authors declare that the research was conducted in the absence of any commercial or financial relationships that could be construed as a potential conflict of interest.

## Publisher’s Note

All claims expressed in this article are solely those of the authors and do not necessarily represent those of their affiliated organizations, or those of the publisher, the editors and the reviewers. Any product that may be evaluated in this article, or claim that may be made by its manufacturer, is not guaranteed or endorsed by the publisher.

## Abbreviations

ABC: ATP-binding cassette transporter A1
AP-1: activator protein-1
CAR: constitutive androstane receptor
C/EBP: CCAAT-enhancer-binding proteins
ChREBP: carbohydrate response element–binding protein
CT: cryptotanshinone
CYP: cytochrome P450
DNL: *de novo* lipogenesis
ERK: extracellular signal-regulated protein kinase
FGF21: fibroblast growth factor 21
FXR: farnesoid X receptor
GLUT-4: glucose transporter 4
JNK: c-JunN-terminal kinases
LA: lithospermic acid
LXR α: liver-X-receptor α
MAPK: mitogen-activated protein kinases
NAFL: non-alcoholic fatty liver
NAFLD: Non-alcoholic fatty liver disease
NASH: non-alcoholic steatohepatitis
NF- κ B: nuclear factor- κ B
OATPs: organic anion transporting polypeptides
PAA: protocatechuic acid
PCA: protocatechuic aldehyde
PPARs: peroxisome proliferator-activated receptors
PXR: pregnane X receptor
RA: rosmarinic acid
RXR α: retinoid X receptor α
SHP: small heterodimer partner
SAA: salvianolic acid A
SAB: salvianolic acid B
SAC: salvianolic acid C
SAD: salvianolic acid D
SREBP-1c: sterol regulatory element-binding protein-1c
SULT: sulfotransferases
STATs: signal transducers and activators of transcription
TCM: traditional Chinese medical
TI: dihydrotanshinone I
TSI: tanshinone I
TSIIA: tanshinone IIA
TSL: tanshinol
UGT: UDP-glucuronosyltransferases.
